# Effect of Different Flours on the Formation of Hydroxymethylfurfural, Furfural, and Dicarbonyl Compounds in Heated Glucose/Flour Systems

**DOI:** 10.3390/foods6020014

**Published:** 2017-02-16

**Authors:** Marta Mesías, Francisco J. Morales

**Affiliations:** Institute of Food Science, Technology and Nutrition (ICTAN), Spanish National Research Council (CSIC), 28040 Madrid, Spain; fjmorales@ictan.csic.es

**Keywords:** flours, Maillard reaction, cereal-based products, hydroxymethylfurfural, furfural, dicarbonyl compounds

## Abstract

Traditional cereal-based foods usually include wheat flour in their formulations; however, the search for new products with new ingredients providing different properties to foods is widely pursued by food companies. Replacement of wheat by other flours can modify both nutritional properties and organoleptic characteristics of the final baked food, but can also impact the formation of potentially harmful compounds. The effect of the type of flour on the formation of furfurals and dicarbonyl compounds was studied in a dough model system during baking that contains water or glucose in order to promote the Maillard reaction and caramelization. The formation of methylglyoxal and glyoxal was significantly reduced in spelt and teff formulations compared to wheat flour formulations, respectively. In contrast, samples formulated with oat, teff, and rye showed a significant increase in the levels of 3-deoxyglucosone. Similarly, spelt and teff formulations presented significantly higher concentrations of hydroxymethylfurfural, and spelt, teff, and rye presented higher concentrations of furfural. Therefore, the formation of process contaminants and undesirable compounds in new food products formulated with different flours replacing the traditional wheat flour should be considered carefully in terms of food safety.

## 1. Introduction

Baked cereal products comprise a wide range of food products subjected to a baking process in which cereal flour is the basic ingredient. Baking involves the application of high temperatures directly on the food product (as high as 260 °C), generally in an oven or heating appliance [1]. The high temperatures applied and the low moisture of the cereal-based products promote the development of different chemical reactions between food components, including the Maillard reaction and caramelization. Both chemical reactions are responsible for the improvement of the textural and organoleptic characteristics of the final baked food by promoting the flavour, colour, and aroma compounds appreciated by the consumers. However, negative changes can occur simultaneously, such as the natural formation of chemical contaminants, that could be mitigated with an adequate selection of the dough recipe [2,3].

Hydroxymethylfurfural (HMF) and furfural are formed as intermediate products of the Maillard reaction and, furthermore, HMF is also generated by the caramelization of sugars at high temperatures [4]. Based on studies in animals, HMF is suspected to have possible mutagenic and genotoxic activities through its metabolism product sulphoxymethylfurfural [5], whereas furfural may lead to hepatotoxicity [6]. On the other hand, dicarbonyl compounds are also generated by the Maillard reaction and caramelization in addition to the lipid oxidation [7]. Dicarbonyl compounds are intermediates of the Maillard reaction, but are also potent promotors of the reaction, since they can interact directly with amino residues or even with other intermediary compounds, leading to the formation of advanced glycation end-products (AGEs) [8]. Dicarbonyl compounds are also responsible for the glycation of several biomolecules in vivo [9], and promoting the formation of circulating AGEs. It has been reported that the consumption of thermally treated foods rich in AGEs could increase the total body AGEs load [8], which is suggested to be implicated in the development of glycation and inflammation associated with the aging process, and complications linked to chronic pathologies such as diabetes, atherosclerosis, and neurodegenerative diseases, among others [10].

Baked cereal products are among the most consumed foods in the Western world [11]. The traditional way to formulate cereal-based foods includes wheat flour in their formulations; however, food companies pursue the search for new products with new ingredients in order to provide different properties to the foods. According to Lovis [12], during the last years there has been a rise in consumer interest in wheat-free foods, due in part to the increase of the celiac disease. When designing new formulas for baked products, the impact of these new ingredients on the nutritional properties of the food and the chemical transformations occurring during the production chain should be assessed. In this sense, many studies have evaluated, among other characteristics, both the technological effects and nutritional properties of bakery products that include flours different from wheat [13,14,15,16]. At this point, the contribution of the new formulations to the intake of HMF, furfural, and dicarbonyl compounds should also be considered and taken into account from a toxicological point of view, since the exposure should not be increased.

The aim of this paper was to study the formation of HMF, furfural, and dicarbonyl compounds, including glyoxal, methylglyoxal, and 3-deoxyglucosone, during the baking step of different flours used by the food industry. It also investigated the effects of the type of flour and the addition of glucose into a dough model system on browning development and the formation of furfurals and dicarbonyl compounds during baking.

## 2. Material and Methods

### 2.1. Chemicals

All chemicals used were of analytical grade and were obtained from Sigma Aldrich (St. Louis, MO, USA). High performance liquid chromatography (HPLC)-grade methanol was from Merck (Darmstadt, Germany).

### 2.2. Samples

Wheat (*Triticum aestivum*), spelt (*Triticum spelta)*, oat (*Avena sativa)*, rye (*Secale cereale)*, and teff (*Eragrostis tef)* flours were purchased from a local food store. Flours (0.5 g) were transferred to screw cap pyrex tubes. Two mL of deionised water and 1 mL of sodium chloride (5 mg/mL) were added to the samples. The mixture was vortexed for 5 min, held at room temperature for 30 min and centrifuged at 1400× *g* for 10 min. The non-absorbed water was discharged and the tube containing the dough with the retained water was closed and baked at 150 °C for 30 min in a forced air convection oven (Memmert UNE 400, Schwabach, Germany). Eight repetitions per type of flour were prepared and combined in two replicates of four samples. Additional experiments were repeated adding 2 mL of glucose (0.15 g/mL) instead of water, together with 1 mL of sodium chloride (5 mg/mL). Eight repetitions per flour were also prepared and combined in two replicates of four samples.

### 2.3. Determination of Moisture

Moisture of flours was determined gravimetrically to a constant weight in an oven at 105 °C for 24 h according to the Association of Official Analytical Chemists (AOAC) method [17].

### 2.4. Determination of Water Holding Capacity (WHC)

Flours (5 g) were placed in a pre-weighed centrifuge tube to which 30 mL of water were added. The mixture was vigorously vortexed for 1 min, held at room temperature for 30 min, and centrifuged at 1400× *g* for 15 min. The non-absorbed water was discharged and the tube was weighed. Water holding capacity according to the weight of samples was calculated by the following formula: ((weight of tube with sample and water retained—weight of tube with sample)/(weight of sample)) × 100. Results are expressed as gram of water retained per 100 gram of flour.

### 2.5. Measurement of pH

Flours or baked dough samples (0.25 g) were mixed with 25 mL of water and vortexed for 3 min. The mixture was held at room temperature for 1 h and centrifuged to separate phases. pH of the supernatant was measured using a CG-837 pH meter (Schott, Mainz, Germany).

### 2.6. Determination of Colour

The measurements were made using a HunterLab Spectrophotometer CM-3500D colorimeter (Hunter Associates Laboratory, Stamford, CT, USA). Three independent measurements of a* (redness), b* (yellowness) and L* (lightness) parameters were carried out on different areas of flours and baked dough samples (with the addition of water or glucose). *E* index was calculated according to the following equation: *E* = (L*^2^ + a*^2^ + b*^2^)^1/2^. Colour difference (∆*E*) was evaluated by comparing the results in baked dough samples to those of initial flours.

### 2.7. Determination of Reducing Sugars (RS)

The reducing sugars content was determined by Miller [18] in the range of 0.25 to 2.0 mg/mL. Results were expressed as g of glucose equivalents/100 g sample.

### 2.8. Determination of HMF and Furfural

HMF and furfural were determined in flours and baked dough samples following the High Performance Liquid Chromatography (HPLC) method described by Mesías et al. [19]. The limit of quantification was determined to be at 0.06 mg/kg and 0.03 mg/kg for HMF and furfural, respectively. Results were expressed as mg/kg sample.

### 2.9. Determination of Dicarbonyl Compounds

Glyoxal (GO), methylglyoxal (MGO), and 3-deoxyglucosone (3-DG) were determined in flours and baked dough samples according to the method of Navarro and Morales [20]. The limit of quantification was determined to be at 0.1, 0.2, and 0.1 µg/g for GO, MGO, and 3-DG, respectively. Results were expressed as µg/g sample.

### 2.10. Statistical Analysis

Statistical analyses were performed using a Statgraphics Centurion XV (Herndon, VA, USA). Unless otherwise indicated, all measurements were performed at least in triplicate. Data was expressed as mean ± standard deviation (SD). Analysis of variance (ANOVA) and the least significant difference (LSD) test were applied to determine differences between means. Differences were considered to be significant at *p* < 0.05. Relationships between the different parameters analysed were evaluated by computing Pearson linear correlation coefficients at the *p* < 0.05 confidence level.

## 3. Results and Discussion

Five cereal flours were selected for the present study, including wheat, rye, oats, and spelt as common flours, as well as teff, an alternative flour used especially in the manufacturing of gluten-free products [21]. Model systems were designed at the maximum water holding capacity of each type of flour and carried out in two set of experiments with the aim of studying the formation of HMF, furfural, and dicarbonyl compounds during baking. In the first experiment, dough was formulated with water and baked for 30 min at 150 °C. The purpose was to examine the contribution of the raw composition of the different flours on the formation of the former compounds since it is known that both recipe composition and thermal treatment are the major factors involved in the extent of the Maillard reaction [22]. In the second experiment, flours were formulated with a solution of glucose with the aim of promoting the Maillard reaction and the caramelization during baking. In this case, the formulation could be considered to be similar to those in a biscuit model system [19]. In both experiments, a solution of sodium chloride (5 mg/mL) was also added into the models, since it is relevant for the dough behaviour and the formation of process contaminants and dicarbonyl compounds during baking of cereal products [19,23].

A proximate composition of the flours is shown in Table 1. Values of pH of the water soluble fraction of the flours were similar, ranging between 6.1 (wheat) and 6.7 (teff). The moisture of flours was in the range of 9.9–10.5 g/100 g. Greater differences were found in the water holding capacity of the flours. Spelt flour retained the 66.0% of its weight, whereas teff and rye flours retained until 116.2% and 124.8%, respectively. Wheat and oat flours showed intermediate values of water retention capacity, 90.7% and 95.8% respectively. The differences in the final water content and the fact that losses of water during the baking process were avoided in the closed tubes make not possible to establish a relationship between the moisture content and the formation of Maillard reaction products. The colour of the samples was measured by a colorimeter in the CIE L*a*b* scale. Due to the initial darker colour of the teff flour, it was significantly different in all the colour parameters respect to the other ones. According to the information provided by the flour manufacturer, wheat and oat flours presented lower values of protein, and spelt and teff flour the highest ones. Regarding reducing sugars content, rye flour showed the highest content (95.5 mg/g), whereas oat flour exhibited the lowest content (11.0 mg/g).

Results of pH values and colour parameters of the baked doughs in the model systems are summarized in Table 2. After baking, the pH was slightly decreased in the models containing water and in a greater extent in the models containing glucose basically due to the formation of formic acid and acetic acid during the extent of the non-enzymatic browning reactions. Sugars and Amadori compounds give rise to the formation of formic and acetic acids. Formic acid is mainly formed in reactions involving disaccharides, while acetic acid is found when monosaccharides are the reactive sugars and form the Maillard reaction [24,25].

The development of the colour is an important indicator of the advance of the Maillard reaction [26]. Regardless the type of flour, doughs formulated with water browned slightly after baking due to the initial content in reducing sugars (Table 2). Moreover, the addition of glucose impacted the colour of the baked dough, and significant differences (*p* < 0.05) were found between samples with added water with respect to those with added glucose. For each formulation, L* values decreased during baking with the addition of water, observing a greater reduction with the addition of glucose. In a similar way, both a* and b* values slightly increased in the baked flours, samples with water exhibiting lower values than those with glucose. ∆*E* ranged from 18.6 to 41.7 in doughs formulated with water, the lowest darkening being displayed in oat and teff. For all the dough model systems, ∆*E* increased with the addition of the sugar (range: 22.5–45.3), thus describing a higher browning process during baking in these samples. In this regard, teff flour showed the lowest ∆*E* after baking in flours with both water and glucose addition, probably because the unbaked flour is already slightly dark. 

**Figure 1:**
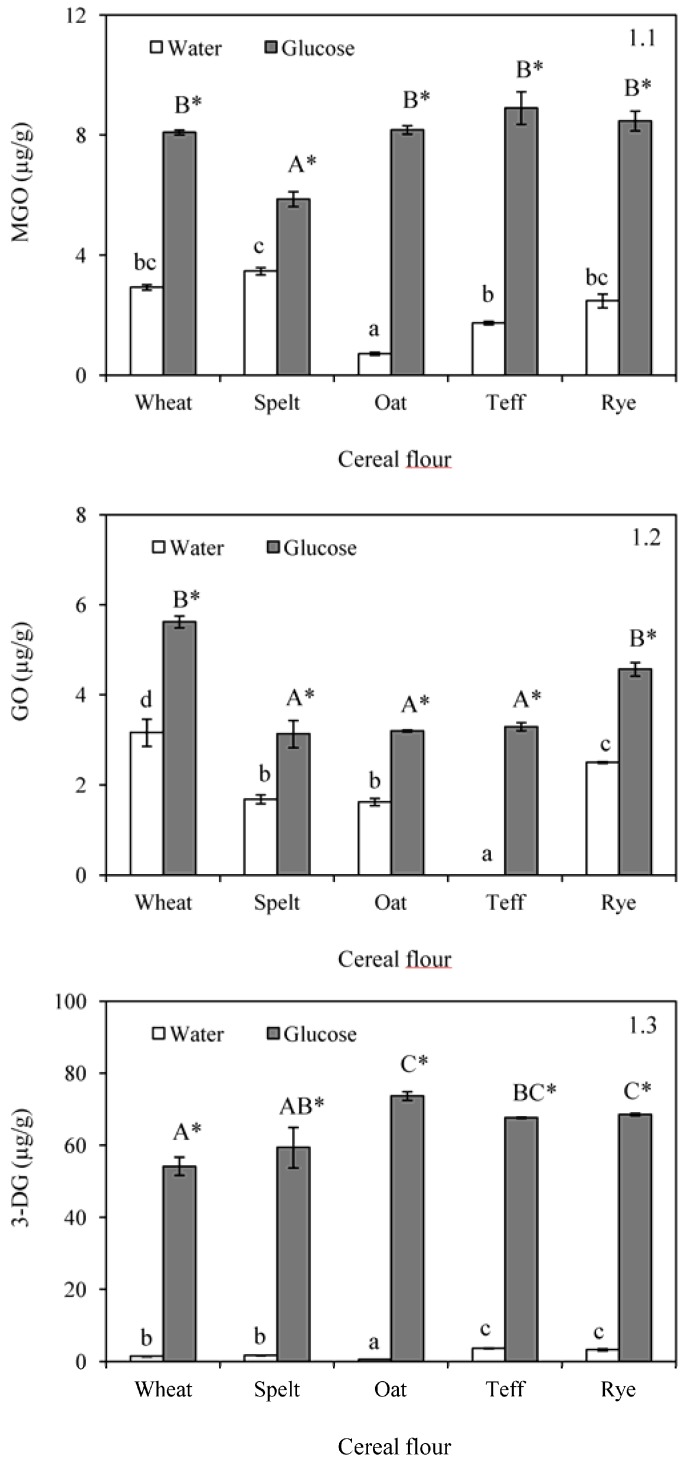
Formation of methylglyoxal (MGO) (**1.1**); glyoxal (GO) (**1.2**); and 3-deoxyglucosone (3-DG) (**1.3**) after baking in doughs formulated with water or glucose and wheat, spelt, oat, teff and rye flour. Results are mean ± standard deviation. a–d: Means with different small letters are significantly different (*p* < 0.05) in baked doughs containing water. A–C: Means with different capital letters are significantly different (*p* < 0.05) in baked doughs with added glucose. *: Asterisk means significant differences (*p* < 0.05) between the same flour when compared glucose and water addition.

The formation of C2, C3, and C6 dicarbonyl compounds was studied in the samples since they are key enhancers of the Maillard reaction, aside from their implication on the generation of flavour compounds. Dicarbonyl compounds were not detected in unbaked flours (Table 1) but the formation was evident in both experiments after baking (Figure 1). In samples formulated with water, values for MGO, GO, and 3-DG ranged between 0.71 and 3.46, not detected −3.16, and 0.63–3.20 µg/kg, respectively. Depending on the content of free amino acids and reducing sugars in the different cereal flours, Maillard reaction or caramelization could be considered as the dominant mechanism in the formation of dicarbonyl compounds during baking of flours. Wheat samples presented intermediate values in MGO and 3-DG, but the maximum concentration of GO. Compared with this flour, the content of dicarbonyl compounds was quite similar in the rest of the samples, with the exception of oats for MGO, which presented the lowest level, and teff for GO, where this chemical product was not present or was below the limit of quantification (0.1 µg/g). The formation of dicarbonyl compounds in a sugar Maillard system is dependent on the pH value. In this regard, only the content of 3-DG was significantly correlated with the pH of the unbaked flours (*r* = 9.9286, *p* = 0.0241). 3-DG also exhibited a positive relationship with the extent of browning after baking (*r* = −0.9121, *p* = 0.0309), which were not observed in MGO and GO.

Levels of dicarbonyl compounds were increased in systems formulated with glucose. In this case, concentrations ranged from 5.86 to 8.90 µg/kg for MGO, from 3.13 to 5.62 µg/kg for GO and from 54.2 to 73.7 µg/kg for 3-DG. The addition of the sugar promoted the formation of MGO mainly in oat and teff, followed by rye and wheat (Figure 1(1.1)). In contrast, spelt displayed the lowest difference between the content of MGO in the dough formulated with water, and this in the dough formulated with glucose, since only an increase of 2.4 µg/kg was measured. Values for GO were slightly higher when glucose was added (increment respect to the systems containing water ranging from 1.4 to 3.3 µg/kg) (Figure 1(1.2)), however, greater differences were observed in the concentrations of 3-DG (Figure 1(1.3)). These results were in agreement with those reported by Kocadağlı et al. [27], who evaluated the content of dicarbonyl compounds in cookies. These authors observed that 3-DG was the predominating compound, except in the cookies from wheat flour, which showed the lowest level compared with other flours. It is known that MGO is formed to a larger extent under Maillard reaction conditions as compared to caramelization [28]; however, 3-DG can be directly formed through dehydration of glucose [23]. This could explain that, with the addition of glucose, the formation of 3-DG is greatly increased compared with the formation of GO and MGO. In this sense, 3-DG concentrations were found to be from 18-fold to even up to 116-fold higher compared with the content of this compound in the baked doughs formulated with water. 

**Figure 2:**
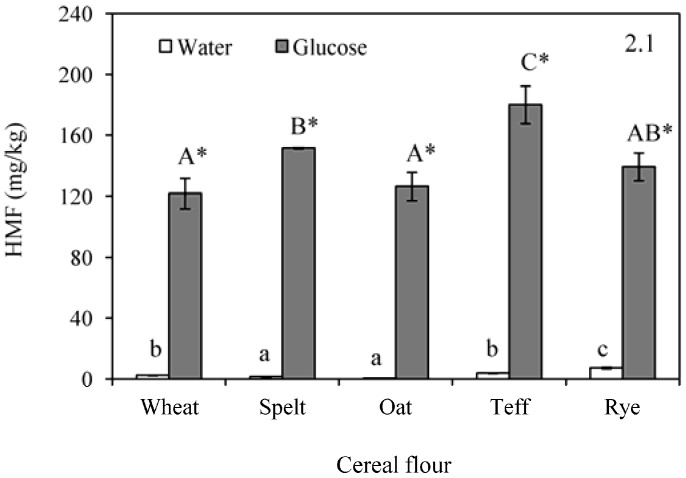

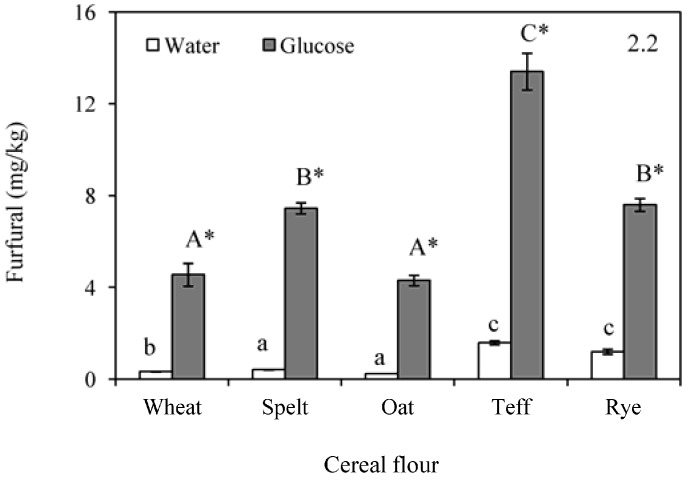
Formation of hidroxymethylfurfural (HMF) (**2.1**); and furfural (**2.2**) after baking in doughs formulated with water or glucose and wheat, spelt, oat, teff and rye flour. Results are mean ± standard deviation. a–c: Means with different small letters are significantly different (*p* < 0.05) in baked doughs containing water. A–C: Means with different capital letters are significantly different (*p* < 0.05) in baked doughs with added glucose. *: Asterisk means significant differences (*p* < 0.05) between the same flour when compared glucose and water addition.

Figure 2(2.1) shows the HMF formation during baking of different flours formulated in water and glucose. Initial flours did not present detectable amount of furfural compounds (Table 1) whereas an increase was observed in baked flours formulated with water (range: 0.5–7.3 mg/kg) and in a greater extent in those formulated with glucose (range: 121.9–180.1 mg/kg). In the system formulated with water, the lowest amount of HMF was found for oat and the highest for rye. These findings could be related to the lowest (11.0 mg/g) and the highest (95.5 mg/g) reducing sugars content in oat and rye flours, respectively (Table 1). However, although the trend was positive (*r* = 0.8601, *p* = 0.0615), no significant correlation was observed between reducing sugars and HMF content in samples formulated with water. The ratio reducing sugars/protein had more influence over the HMF content and a significant correlation was found among these parameters (*r* = 0.9079, *p* = 0.0331). This fact shows the contribution of both components, reducing sugars and protein, on the formation of HMF, probably coming from the Maillard reaction. As expected, HMF also correlated with the browning development after baking, displaying a significant negative relation with the E value (*r* = −0.9368, *p* = 0.0189). HMF was highly affected by the addition of glucose, which promotes both the Maillard reaction and the caramelization. These findings agree with those of Gökmen et al. [29], who reported that the effect of glucose on HMF formation is much greater than the effect of other sugars like sucrose. In this condition, the lowest value was notable in wheat and the highest one in teff, which was highlighted due to the significantly higher concentrations. The increase of the sugar content by the addition of glucose could explain the absence of relation between HMF and the levels of sugars in the initial flours. Protein content and HMF were not correlated, which could suggest that a high contribution of the caramelization to the HMF generation. The pH values of the samples could also be involved in the HMF formation. In general, lowering the pH increases the tendency of the HMF formation in cereal-based foods during baking, and this effect can be more pronounced for the dough comprising glucose [29], which is in agreement with the results of the present study. 

Furfural showed the same behaviour as HMF during baking (Figure 2(2.2)). Furfural concentration in unbaked samples was below the quantification limit of 0.03 mg/kg (Table 1). After baking, samples containing water exhibited a range of furfural concentration between 0.2 and 1.6 mg/kg, being related with the browning development, as shown by the significant correlation (*p* < 0.05) with the L*a*b* parameters (*r* = −0.9072, *p* = 0.0335 for L*; *r* = 0.8937, *p* = 0.0409 for a*; *r* = −0.9463, *p* = 0.0032 for b*). It was also found that the flour influenced the amount of furfural generated. Wheat had intermediate values, being surpassed by teff and rye. The addition of glucose also displayed a strong influence on furfural formation in baked dough system models. Wheat and oats presented increments of around 4.2 mg/kg, spelt and rye between 6.4 and 7.0 mg/kg, and teff, again, manifested the highest differences (11.8 mg/kg). Furfural formation can also be related with the pH of the samples. In this regard, a significant relationship was found between the pH in unbaked flours with the furfural generated in baked doughs with water (*r* = 0.9802, *p* = 0.0033) and between pH and furfural in baked doughs with glucose (*r* = 0.9326, *p* = 0.0208). A positive correlation (*r* = 0.9738, *p* = 0.0051) was also found between HMF and furfural in the baked samples containing glucose, which corroborates the parallel development of the Maillard reaction products.

It is known that dicarbonyl compounds are highly reactive compounds which can promote the advance of the Maillard reaction towards the formation of dietary AGEs after the reaction with amino residues or even other intermediary compounds as HMF by dehydration [8]. In this line, Navarro and Morales [20] reported a positive relationship between HMF and 3-DG in cookies. In the present study, a significant relationship was found between 3-DG and furfural content (*r* = 0.9737, *p* = 0.0051) whereas, although no significant, the trend was also positive among 3-DG and HMF (*r* = 0.8220, *p* = 0.0877). In contrast, HMF did not significantly correlate with either GO or MGO, in agreement with the observations found by Arribas-Lorenzo and Morales [28] in commercial cookies. 

## 4. Conclusions

The composition of the flour will determine the formation of furfurals and C2, C3, and C6 dicarbonyl compounds in the baked dough. During baking, there is a concomitant formation of desirable and undesirable compounds from the non-enzymatic browning reactions that will impact on the consumer perception and the chemical safety of the product. The food industry commonly identifies the functional performance of any ingredient on a cereal-based formulation in terms of consumer acceptability and shelf-life, but food safety issues should be considered as well. In this sense, there is a growing interest for wheat-free foods. Replacement of wheat flour by spelt, oat, rye, and teff flours could be an alternative, maintaining an adequate nutritional value, but could have an impact the levels of potentially harmful compounds. Compared with wheat flour formulations, spelt and teff significantly reduced the formation of methylglyoxal and glyoxal, respectively. However, 3-deoxyglucosone formation is significantly increased in samples formulated with oats, teff, and rye. In a similar way, HMF was significantly increased in spelt and teff formulations and furfural for spelt, teff and rye. Therefore, the replacement of wheat flour in bakery products for the development of new food products could present side effects related to the formation of process contaminants and undesirable compounds that should be considered carefully in terms of food safety.

## Figures and Tables

**Table 1:** Characterization of flours in terms of pH value, moisture, water holding capacity, L*a*b* colour scale, colour *E* index, reducing sugars content, protein, hydroxymethylfurfural, furfural, methylglyoxal, glyoxal, and 3-deoxyglucosone.

	Wheat	Spelt	Oat	Teff	Rye
pH	6.1 ± 0.1a	6.2 ± 0.1a	6.2 ± 0.1a	6.7 ± 0.0b	6.5 ± 0.2b
Moisture (%)	10.3 ± 0.0b	10.5 ± 0.1b	9.9 ± 0.1a	9.9 ± 0.0a	10.0 ± 0.1a
WHC (%)	90.7 ± 1.5b	66.0 ± 1.4a	95.8 ± 1.6c	116.2 ± 1.2d	124.8 ± 1.8e
a*	0.1 ± 0.0a	0.1 ± 0.0a	0.3 ± 0.0a	4.3 ± 0.2b	0.1 ± 0.0a
b*	8.4 ± 0.2b	8.9 ± 0.4bc	9.2 ± 0.6c	10.3 ± 0.3d	7.7 ± 0.1a
L*	91.4 ± 0.6d	89.0 ± 0.7c	87.0 ± 0.1b	68.3 ± 0.7a	87.2 ± 0.3b
*E* index	91.8 ± 0.5c	89.4 ± 0.7c	87.5 ± 0.1b	69.2 ± 0.7a	87.5 ± 0.2b
RS (mg/g)	30.0 ± 0.2c	36.6 ± 0.5d	11.0 ± 0.2a	26.7 ± 0.3b	95.5 ± 2.0e
Protein (g/100 g) ^1^	12.2	14.6	11.0	13.3	12.0
HMF (mg/kg)	<LOQ	<LOQ	<LOQ	<LOQ	<LOQ
Furfural (mg/kg)	<LOQ	<LOQ	<LOQ	<LOQ	<LOQ
MGO (µg/g)	<LOQ	<LOQ	<LOQ	<LOQ	<LOQ
GO (µg/g)	<LOQ	<LOQ	<LOQ	<LOQ	<LOQ
3-DG (µg/g)	<LOQ	<LOQ	<LOQ	<LOQ	<LOQ

^1^ Protein content has been provided by the flour manufacturer. WHC: water holding capacity. RS: reducing sugars. HMF: Hydroxymethylfurfural. MGO: Methylglyoxal. GO: Glyoxal. 3-DG: 3-deoxyglucosone. LOQ: Limit of quantification. CIELAB parameters: L* defines lightness, a* denotes the red/green value and b* the yellow/blue value. Results are mean ± standard deviation. Different letters in the same row mean significant differences (*p* < 0.05).

**Table 2:** Colour parameters and pH of baked doughs formulated with water or glucose and wheat, spelt, oat, teff and rye flour.

	Wheat		Spelt		Oat		Teff		Rye	
	Water	Glucose	Water	Glucose	Water	Glucose	Water	Glucose	Water	Glucose
pH	5.6 ± 0.1a	4.9 ± 0.1A *	6.0 ± 0.0b	5.4 ± 0.0B *	6.2 ± 0.0b	5.2 ± 0.1B *	6.3 ± 0.1b	6.1 ± 0.0C	5.7 ± 0.2a	5.4 ± 0.0B
L*	54.7 ± 1.1b	44.4 ± 0.6B *	53.4 ± 2.6b	43.4 ± 2.3B *	60.4 ± 0.9c	42.2 ± 1.3AB *	45.9 ± 0.9a	48.7 ± 2.0C	44.6 ± 1.4a	39.5 ± 1.3A *
a*	3.6 ± 0.4a	6.7 ± 0.6B *	3.6 ± 0.5a	4.7 ± 0.6A	3.1 ± 0.6a	8.7 ± 1.1C *	4.6 ± 0.2b	6.1 ± 0.6B *	3.7 ± 0.6a	6.5 ± 0.2B *
b*	14.6 ± 1.5c	15.4 ± 2.0BC	12.4 ± 1.9b	8.0 ± 1.4A *	17.0 ± 1.4c	17.5 ± 2.0C	7.3 ± 0.9a	12.2 ± 1.6B *	9.5 ± 1.3ab	13.3 ± 1.2B *
∆*E*	35.0 ± 0.5c	44.2 ± 0.5C *	34.5 ± 2.2c	45.0 ± 1.9C *	24.6 ± 0.5b	40.9 ± 1.4B *	18.6 ± 1.3a	22.5 ± 0.3A *	41.7 ± 1.2d	45.3 ± 1.3C *

Results are mean ± standard deviation. a–d: Means with different small letters are significantly different (*p* < 0.05) in baked dough model systems formulated with water. A–C: Means with different capital letters are significantly different (*p* < 0.05) in baked dough model systems formulated with glucose. *: Asterisk means significant differences (*p* < 0.05) between the dough formulated with the same flour when compared glucose and water system. ∆*E*: *E* flour—*E* baked dough model system containing water or glucose.
